# Hematological and serum biochemical profile in cattle experimentally infected with foot-and-mouth disease virus

**DOI:** 10.14202/vetworld.2020.426-432

**Published:** 2020-03-09

**Authors:** S. Saravanan, V. Umapathi, M. Priyanka, M. Hosamani, B. P. Sreenivasa, B. H. M. Patel, K. Narayanan, Aniket Sanyal, S. H. Basagoudanavar

**Affiliations:** Indian Council of Agricultural Research-Indian Veterinary Research Institute, Bengaluru, Karnataka, India

**Keywords:** biochemical profile, experimentally infected indigenous and crossbred calves, foot-and-mouth disease, hematological profile

## Abstract

**Background and Aim:**

Foot-and-mouth disease (FMD) is an acute viral infection affecting cloven-hoofed animals causing vesicular erosions in the oral cavity and interdigital space. The present study was undertaken to ascertain the time-dependent changes in clinical, hematological, and biochemical profiles in different breeds of cattle following experimental infection.

**Materials and Methods:**

The animals were inoculated with 1.0×10^4^ 50% bovine tongue infectious dose (BTID_50_) by intradermolingual route. Clinical signs were observed, and blood/serum samples were collected at different time intervals.

**Results:**

The white blood cell count declined sharply on days 7-13 and recovered on day 14 post-FMD infection. Biochemical analysis of serum markers for vital organ profile revealed no marked damage. However, a significant increase in blood urea nitrogen (BUN) value indicated pre-renal azotemia. Transient hyperthyroidism was indicated by the rise in T3 and T4 that can be correlated with a decrease in triglyceride and total cholesterol levels. In the cardiac damage assessment study, a distinct breed difference was observed wherein Malnad Gidda calves showed no cardiac damage.

**Conclusion:**

Except thyroid profile, BUN, and creatine kinase-myocardial band, all other serum biochemical parameters showed no significant abnormalities, whereas lymphopenia is the only hematological change and it is suggested that effective ameliorative measures should be targeted mainly on the feed/water intake, thyroid gland, and the level of lymphocytes.

## Introduction

Foot-and-mouth disease (FMD) is the most economically important viral disease of cattle, caused by FMD virus (FMDV) and is characterized by fever, depression, anorexia, and excessive stingy or foamy salivation with vesicles or blisters appearing on the tongue. Some infected animals remain asymptomatic, but they carry the virus and can transmit it to other animals. The disease is generally not fatal (mature livestock) but increases the risk of abortion (pregnant animals) and of mortality (young livestock). FMD leads to reduced productivity and requires increased expenditures on feed, medication, and shelter [1]. The economic losses caused by the disease are mainly due to losses in milk production and reduction in the working capacity of draught-animals. In addition, milk and milk products, meats and hides are not accepted by the disease-free importing countries causing a reduction in the export potential of the livestock industry [2,3].

The initial diagnosis of the disease is usually done on the basis of clinical signs. In endemic regions, the partial signs are generally overlooked due to natural or vaccinal immunity [4]. The level of certain serum metabolites indicates the different disease conditions and degree of stress in the animal [5]. However, scanty literature is available on the effect of experimental FMD infection on blood and serum profile of cattle, which is important for formulating the ameliorative measures to reduce the biotic stress on the animal due to the infection. In addition, an Indian native breed, Malnad Gidda, is believed to be tolerant of many infectious diseases including FMD; however, no systematic study has been carried out to establish the exactness of this perception [6,7].

Based on the above-mentioned facts, the present study has been undertaken to elucidate the effect of FMD infection on certain clinical, hematological, and serum biochemical parameters in few breeds of cattle during the course of the disease after experimentally infecting the calves.

## Materials and Methods

### Ethical approval

The animal experimental protocol was approved by the Institutional Animal Ethics Committee of ICAR- Indian Veterinary Research Institute, Bengaluru and carried out according to the guidelines of the Committee for the Purpose of Control and Supervision of Experiments on Animals, Ministry of Environment, Forests and Climate Change, Government of India.

### Experimental animals

Bull calves of 6-9 months of age (n=4/breed, namely, Malnad Gidda, Hallikar, and Holstein Friesian cross breed) with a history of no previous infection and not vaccinated for FMD, were used in the study. They were dewormed and kept under quarantine and acclimatization for 4 weeks. All the animals were negative for non-structural protein antibodies [8]. They were also seronegative (virus neutralization VN titer of ≤8) for FMDV structural antigens [9].

### Virus infection

A bovine-derived FMDV serotype O/IND/R2/75 was used for experimental infection. Four calves per breed were inoculated with 1.0×10^4^ 50% bovine tongue infectious dose (BTID_50_) of virus suspended in 0.2 mL media by intradermolingual route, in containment animal facility. Observations on day 0 (the day of infection) were made before inducing experimental FMDV infection.

### Blood/serum collection

Blood samples of 10 mL were collected by jugular venipuncture in a red vacutainer containing clot activator (BD, Franklin, USA) and serum was separated by centrifugation at 3000× *g* for 15 min and stored at −80°C until assay for serum biochemical parameters. For hematological analysis, 2 mL blood sample was collected in a green vacutainer containing heparin as an anticoagulant (BD, Franklin, USA).

### Complete blood count analysis

Complete blood count analysis was carried out using a double chamber hematology analyzer (Unitron Biomedicals, India).

### Gross observation for clinical signs

Calves were observed for the onset, progress, and remission of secondary lesions in the foot at 24 h interval for 30 days post-infection. The foot lesion was scored on a scale of 1-4 based on the number of limbs involved. Rectal temperature was recorded from day 0 to 10 post-infection on a daily basis.

### Serum biochemistry

Total serum protein, albumin, triglycerides, and total cholesterol were estimated with commercial kits using bench-top semi-automatic biochemistry analyzer (Erba Chem 7, Germany). The concentration of globulin and albumin to globulin (A: G) ratio was derived from total protein and albumin. Cardiac damage was assessed by the immunoinhibition method using the creatine kinase-myocardial band (CK-MB) kit (Coral Clinical Systems, India). Liver function was evaluated by aspartate transaminase (AST), alanine transaminase (ALT), and gamma-glutamyl transferase (GGT) levels, while azotemia was assessed by blood urea nitrogen (BUN) and creatinine. The concentration of triiodothyronine (T3), thyroxine (T4), and thyroid-stimulating hormone was estimated by standard kit.

### Electrocardiography (ECG)

Electrocardiography (ECG) was recorded in bipolar base apex lead (CARDIART 6208, BPL) using limb lead I on day 0 (pre-infection), 1-5 days, and 21-day post-infection. ECG was recorded in standing position by placing the rubber mat underneath the animal without any tranquilizer or sedative. Alligator type electrodes were attached to skin after cleaning it with ethanol and applying electrocardiographic jelly. The positive electrode of the lead I was attached to the skin of the fifth intercostal space just caudal to olecranon process and negative electrode on jugular furrow about lower one-third of the left side of the neck. ECG was recorded in a single channel with a paper speed of 25 mm/s and calibration of 10 mm equal to 1 mV.

### Statistical analysis

The sample size for infected samples comprised 12 animals (n=4 per breed) for the parameters observed till day 10 post-infection. However, for later days, it was n=11, as one Malnad Gidda calf died on 11 dpi. Two-way ANOVA with Tukey’s *post hoc* test was done for viremia, CK-MB, and one-way repeat measure ANOVA was done with orthogonal contrast (pre-infection vis-à-vis post-infection) using Tukey’s *post hoc* test. Data were analyzed by GraphPad Prism 6.0. The value of p=0.05 or less was considered statistically significant.

## Results

**Figure-1:**
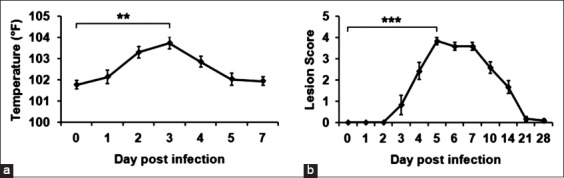
Progress of clinical signs in calves experimentally infected with the foot-and-mouth disease virus. (a) Rectal temperature (mean±standard error of the mean [SEM]) during the course of acute foot-and-mouth disease virus (FMDV) infection. The elevated temperature was observed on day 3 post-infection and subsided by day 5 post-infection, **p<0.01. (b) Foot lesion score (mean±SEM) during the course of FMDV infection. The foot lesions peaked on day 5 post-infection, and disappeared by 21 days post-infection, ***p<0.001.

The onset of pyrexia (>103° F) on day 2 and foot lesions on day 3 indicates that the animals were infected with FMDV (O/R2/75). Pyrexia and lesions reached peak values on days 3 and 5 post-infection, whereas remission was observed on day 5 and 21-day post-infection, respectively (Figure-1a and b).

**Figure-2:**
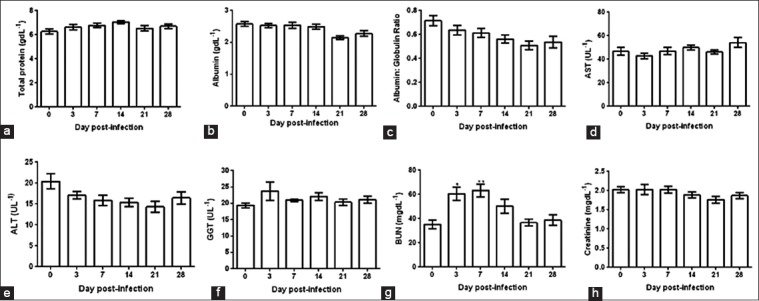
Serum biochemical profile of calves infected with foot-and-mouth disease virus (FMDV). (a-h) Indicate serum total protein, albumin, A: G ratio, aspartate transaminase, alanine transaminase, gamma-glutamyl transferase, blood urea nitrogen, and creatinine, respectively. The data were analyzed by one-way repeat measure ANOVA with Bonferroni *post hoc* test. Orthogonal contrast was used to compare the mean of day 0 with other days post-FMDV. Multiplicity adjusted p-value was calculated to minimize the alpha error.

The serum total protein, albumin, AST, ALT, GGT, and creatinine were within the physiological range up to 1 month post-infection. However, BUN showed a significant (p *<* 0.05) increase during days 3-14 post-infection (Figure-2a-h).

**Figure-3:**
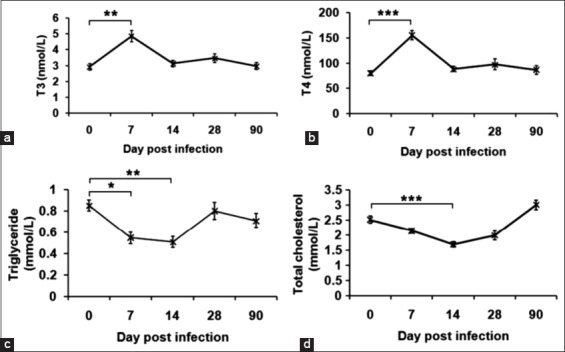
Thyroxine and lipid profile of calves infected with foot-and-mouth disease virus (FMDV). (a) Changes in the levels of triiodothyronine (T3) during FMDV infection. (b) Changes in the levels of thyroxine (T4) during FMDV infection. (c) Changes in the levels of triglyceride during FMDV infection. (d) Changes in the levels of total cholesterol during FMDV infection. There was a significant increase in the levels of T3 and T4 on 7 days post-infection, associated with a decrease of triglyceride and total cholesterol on 7-14 days post-infection, *p<0.05; **p<0.01; ***p<0.001.

Although there was no breed difference, a marked elevation (p<0.05) of T_3_ and T_4_ was observed on day 7 post-FMD as compared to pre-infection (Figure-3a and b). The concentration was above the upper physiological limit, which is likely due to the release of the stored thyroxine in response to viral-induced damage to follicular cells of the thyroid gland. Transient hyperthyroidism is partly supported by decreased concentrations of serum triglyceride and cholesterol on days 7-14 post-FMDV in the bull calves (Figure-3c and d).

**Supplementary Figure-1:**
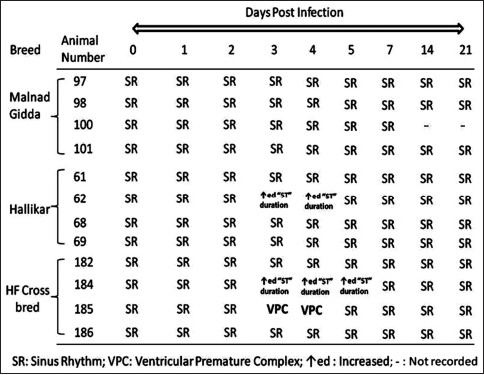
Pattern of ECG changes observed in cattle of three different breeds during experimental FMDV infection.

**Figure-4:**
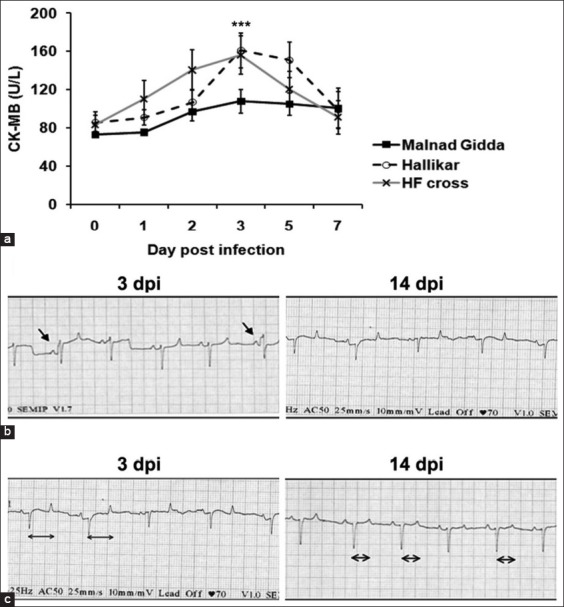
Kinetics of serum cardiac marker creatine kinase-myocardial band (CK-MB) and ECG of foot-and-mouth disease virus (FMDV) infected calves. (a) Temporal level of serum CK-MB during acute FMDV infection. The CK-MB release was significantly low in Malnad Gidda on day 3 post-infection compared to Hallikar and HF crossbred animals. ***p<0.001. (b) Representative electrocardiogram showing interpolated ventricular premature complexes in a HF crossbred calf (indicated by arrow mark) on 3 days post-infection. Sinus rhythm was re-established by 14 days post-infection. (c) Representative electrocardiogram showing increased “ST” duration (0.40 s) in a HF crossbred calf (indicated by horizontal arrow mark) on 3 days post-infection. Sinus rhythm was re-established by 14 days post-infection.

Since FMDV is known to induce myocarditis in calves, CK-MB was estimated in serum samples to assess the severity of damage from pre-infection to day 7 in the experimental calves (Figure-4a). A significant breed effect (p<0.001) was observed; the concentration of CK-MB was markedly low in Malnad Gidda on days 2 and 3 post-infection. In contrast, the Hallikar and crossbred HF calves had higher CK-MB levels, indicative of myocardial damage. This is supported by the pattern of ECG changes observed in all the three different breeds during experimental infection (Supplementary Figure-1). Normal sinus rhythm was observed in Malnad Gidda calves throughout the observation period (0-21 days post-infection), whereas one Hallikar and two crossbred HF calves showed arrhythmias like interpolated ventricular premature complexes (Figure-4b left panel) and increase in the “ST” duration (0.40 s) (Figure-4c left panel) on day 3, 4, and 5 post-infection. Reestablishment of normal sinus rhythm and ECG parameters (“ST duration’’ reduced to 0.30 s) was observed by 14 days post-infection (Figure-4b right panel and Figure-4c right panel).

**Figure-5:**
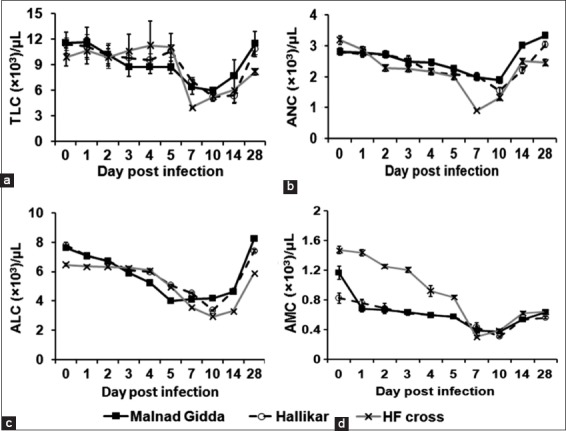
Temporal levels of white blood cell subpopulations in foot-and-mouth disease virus (FMDV) infection in calves. (a) The counts of total leukocytes. (b) The counts of neutrophils. (c) The counts of lymphocytes. (d) The counts of monocytes. A significant decline in the cells was observed during days 7-10 post-FMDV infection. The decline in WBC counts was relatively less in the Malnad Gidda and Hallikar breed calves compared to HF crossbred animals.

Total leukocyte count revealed a significant decrease (p<0.05), which was apparent during days 7-14 post-infection indicating leukopenia (Figure-5a). The neutrophil count showed a significant decrease (p<0.05) on days 7 and 10 that returned to the pre-infection level by day 14 post-infection (Figure-5b). A significant decrease (p<0.05) in lymphocyte consistent with lymphocytopenia was recorded between days 7 and 14 post-infection (Figure-5c). Although a similar trend was observed for monocytes, monocytopenia persisted till day 28 post-infection (Figure-5d).

## Discussion

Despite the widespread use of antimicrobials and vaccinations, infectious diseases pose a major threat to livestock health. Therefore, research focused on the influence of host genetic variations in susceptibility to infectious diseases including FMD, is gaining relevance, to aid develop strategies for selective breeding. In addition, the stress posed by the viral infection on the animal result in production loss. An understanding of the changes in the pattern of clinical, serum biochemical, and hematological parameters during the course of FMD infection is important in developing therapeutic approaches for circumventing the stress and thereby reducing the negative effect of the infection in the animal. In addition, as mentioned elsewhere, a few Indian native breeds are said to be relatively tolerant of many infectious diseases including FMD. Therefore, the present study was aimed at studying the changes in some clinical, biochemical, and hematological parameters in different breeds of cattle experimentally infected with FMDV. Although hematological and serum biochemical changes have been reported in different species of animals [10-12], studies on time-dependent changes during the course of infection including the acute phase of the disease are not available. We also preferred to induce experimental infection as it is difficult to get different breeds of animals with similar age following natural infection in a specific geographical region.

Therefore, in this study, we evaluated the clinical, serum biochemical, and hematological responses during the course of experimental FMDV infection in Malnad Gidda, Hallikar (another Indian native breed), and Holstein Friesian crossbred calves.

The onset of pyrexia started by day 1 in all the breeds, while peak pyrexia was observed on day 2 in Malnad Gidda and day 3 in Hallikar and crossbred HF. Peak lesions appeared on 5^th^ day in all breeds while the healing of wound was early by 21 days post-infection in Malnad Gidda and Hallikar and 28 days in crossbred HF indicating Indian native breeds have better healing ability from infections.

The serum biochemistry showed no significant change in total protein, albumin, and A: G ratio. This is in contrast to a significant decrease in serum total proteins observed after natural infection in the previous studies [11-13]. Furthermore, liver function was not affected as evidenced by unaltered AST, ALT, and GGT levels. However, normocreatinemia with increased BUN on days 3-7 was observed, which could be due to pre-renal azotemia caused by anorexia and dehydration, fever, increased catabolism, and tissue damage [14]. Since the thyroid gland is one of the predilection sites of FMDV where viral replication and persistence are reported [15], we estimated thyroxine levels in the FMDV infected animals. Elevated concentration of serum T_3_ and T_4_ on 7 days post-infection indicates the release of the stored hormones due to FMDV induced damage to follicular cells of thyroid rather than altering the negative feedback regulation. Further, a significant reduction in the serum triglyceride and total cholesterol on 7 days post-infection is likely due to the transient elevation of thyroid hormones, as reported earlier [16]. Notably, no significant variation between the native and HF crossbred cattle was observed with respect to these biochemical parameters. The CK-MB has been used as a diagnostic biomarker indicating injury in the myocardium [17].

Interestingly, the alteration in the CK-MB level in Malnad Gidda was insignificant compared to Hallikar and HF crossbred calves. This suggested that Malnad Gidda suffered less myocardial tissue damage during FMDV infection. Hallikar and HF crossbred calves had significantly elevated CK-MB levels, which were corroborated by the typical arrhythmic changes such as preventricular complexes and prolonged ST waves observed in ECG on day 3 post-infection indicating myocardial damage. The ECG recorded on day 3 for Malnad Gidda and day 14 post-infection for all animals showed normal sinus rhythm. However, Aktas *et al*. [18] observed tachypnea, tachycardia, and gallop rhythm in naturally infected calves with myocarditis and cardiac troponin-I and AST were significantly higher, but the levels of CK-MB and lactate dehydrogenase were not.

With regard to hematological analysis, there was a significant decrease in total leukocyte, absolute counts of neutrophil, lymphocyte, and monocytes in FMDV infected calves. This is in agreement with previously reported leukopenia in cattle and swine during the virus infection [10,19]. Although there was no significant difference between breeds tested, it was observed that the trend of decrease in leukocyte levels was comparatively less in Malnad Gidda. Considering the positive association between virus load and leukopenia during acute viral infections [20], it is possible that relatively less leukopenia observed in Malnad Gidda calves could be due to early clearance with less virus load in the system [21].

## Conclusion

To sum up, the FMD infected animals showed significant clinical signs during the course of infection, and the animals completely recovered as early as 21 and 28 days in case of native breeds and HF cross-breed animals, respectively. Except for thyroid profile, BUN, and CK-MB, all other serum biochemical parameters showed no significant abnormalities. Lymphopenia is the only hematological change. Based on these observations, it is suggested that effective ameliorative measures should be targeted mainly on the feed/water intake, thyroid gland, and the level of lymphocytes. As far as the breed difference is concerned, Malnad Gidda calves showed relatively less impact on myocardial changes and leukopenia. However, further studies on immunological and molecular pathways are needed to confirm their relative tolerance for FMD.

## Authors’ Contributions

SHB, AS and VU conceived the problem, designed the work, and finalized the manuscript. SS and MH carried out animal and laboratory experiments, MP and BHMP carried out animal experiments including ECG work, KN and BPS analyzed and interpreted the data. All authors read and approved the final manuscript.
